# Vibrating Makes for Better Seeing: From the Fly’s Micro-Eye Movements to Hyperacute Visual Sensors

**DOI:** 10.3389/fbioe.2014.00009

**Published:** 2014-04-28

**Authors:** Stéphane Viollet

**Affiliations:** ^1^Aix-Marseille University, CNRS, ISM UMR 7287, Marseille, France

**Keywords:** hyperacuity, eye, micro-movements, fly, visual sensors, robots, micro-scanning

## Abstract

Active vision means that visual perception not only depends closely on the subject’s own movements, but that these movements actually contribute to the visual perceptual processes. Vertebrates’ and invertebrates’ eye movements are probably part of an active visual process, but their exact role still remains to be determined. In this paper, studies on the retinal micro-movements occurring in the compound eye of the fly are reviewed. Several authors have located and identified the muscles involved in these small retinal movements. Others have established that these retinal micro-movements occur in walking and flying flies, but their exact functional role still remains to be determined. Many robotic studies have been performed in which animals’ (flies’ and spiders’) miniature eye movements have been modeled, simulated, and even implemented mechanically. Several robotic platforms have been endowed with artificial visual sensors performing periodic micro-scanning movements. Artificial eyes performing these active retinal micro-movements have some extremely interesting properties, such as hyperacuity and the ability to detect very slow movements (motion hyperacuity). The fundamental role of miniature eye movements still remains to be described in detail, but several studies on natural and artificial eyes have advanced considerably toward this goal.

## 1 Introduction

In their discussion about hoverflies visual flight control, Collett and Land (1975) argued that an animal might be capable of dealing with a constantly moving image on the basis of head and body movements, but its behavior would be different in this case. However, retinal micro-movements do occur in hoverflies and flies, due to the presence of a muscle in charge of moving the retina (Burtt and Patterson, 1970).

Many studies have been published on the micro-movements produced by human eyes (for a review of the literature on micro-saccades, see Rolfs, 2009) their characteristics (their amplitude, frequency, etc.), their role, their neural basis, etc. However, much less attention has been paid so far to this topic in the case of invertebrates. The small amplitude eye movements known to occur in humans have been classified in three categories: tremor, drift, and micro-saccades (Carpenter, 1988). Ocular micro-movements are also known to occur in invertebrates, since scanning micro-movements have been observed in several invertebrates such as crabs (Burrows and Horridge, 1968; Sandeman, 1978), arachnids (Land, 1969), mollusks (Land, 1982; Kaps and Schmid, 1996), and flies (see Retinal Micro-Movements in the Fly’s Compound Eye: A Review). All these studies are an endless source of inspiration for developing new sensing techniques such as those presented in Section “Bio-Inspired Visual Sensors Mimicking Animals’ Micro Eye Movements” of this review. However, as discussed by Webb (2000) and recalled by De Rossi and Pieroni (2013), the bio-inspired sensors presented here can also be used to test the validity of biological hypotheses.

The main role of micro-eye movements is certainly to generate temporal changes serving mainly to prevent the occurrence of the visual adaptation (fading), which normally occurs when images are perfectly stabilized on the retina. However, recent findings on humans (Ko et al., 2010; Kuang et al., 2012; Martinez-Conde et al., 2013) tend to prove that micro-saccades also contribute importantly to the processes involved in hyperacuity. An interesting suggestion has been made by Ahissar and Arieli (2012), according to which the temporal encoding and decoding of the visual signals resulting from fixational eye movements may result in highly acute vision, as long as some assumptions about the velocity of the eye movements prove to be true.

Hyperacuity, which is defined in detail in Section “Some Findings on Visual Acuity,” depends on the ability to locate an object with greater accuracy than that imposed by the photoreceptor’s pitch. The artificial micro-scanning sensors, depicted in Section “Bio-Inspired Visual Sensors Mimicking Animals’ Micro Eye Movements,” driving robots periodic eye micro-movements certainly do promote hyperacuity.

## 2 Retinal Micro-Movements in the Fly’s Compound Eye: A Review

**Figure 1:**
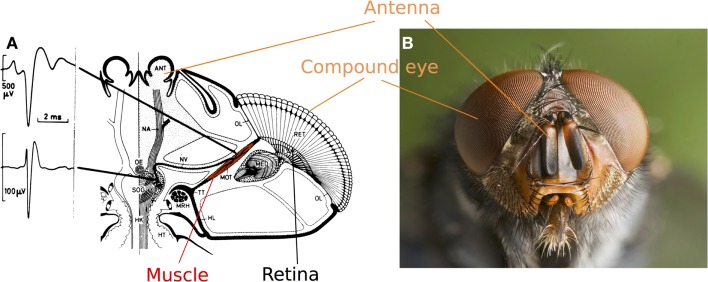
**(A)** Top view of a fly’s head showing the orbito-tentorialis muscle (MOT in red) attached to the back of the head (the fixed part: TT) and the base of the retina (the moving part: RET). The two spikes recorded (one generated by the nerve and one by the MOT) show that extracellular recordings can be used to record the activity of this muscle. Adapted from (Hengstenberg, 1972). **(B)** Head of a *Calliphora vomitoria* (Picture: J. J. Harrison, Wikimedia commons).

In their 1965 study, Kuiper and Leutscher-Hazelhoff described what they called clock-spikes occurring in the third ganglion layer of the optic lobe (Kuiper and Leutscher-Hazelhoff, 1965). Although the firing rate was found to be very consistent (50 Hz) whatever the type of stimulus used (electric light, flash light, etc.), it increased with the temperature. As the authors thought it was unlikely that flies might be equipped with a built-in thermometer, they suggested that “clock-spikes” might provide the visual system with inputs serving to locate objects, but the insect had to be aware of its velocity and the line of sight of the ommatidium of interest. A few years later, by placing a micro-electrode (30 μm in size) in contact with a specific muscle in the blowfly’s head called the orbito-tentorialis muscle (MOT), which is attached to the back of the head (the fixed part) and the base of the photoreceptor layer (the moving part), Burtt and Patterson (1970) established that the MOT is responsible for generating these clock-spikes (see Figure 1).

Although Kuiper and Leutscher-Hazelhoff did not observe any responses to light in the MOT, Burtt and Patterson recorded a marked change in the firing rates in response to sudden light variations, although no rhabdomere movements were recorded under steady light conditions (Burtt and Patterson, 1970; Patterson, 1973b). Preliminary studies showed that the activity of the MOT might be correlated with the movement of a pattern crossing the visual field (Hengstenberg, 1971, 1972). However, the movements of the photoreceptors relative to the lens and the correlations possibly existing between locomotion and photoreceptor movements still remained to be investigated (see Section “Recording the Fly’s Photoreceptor Movements”).

### 2.1 Recording the fly’s photoreceptor movements

**Figure 2:**
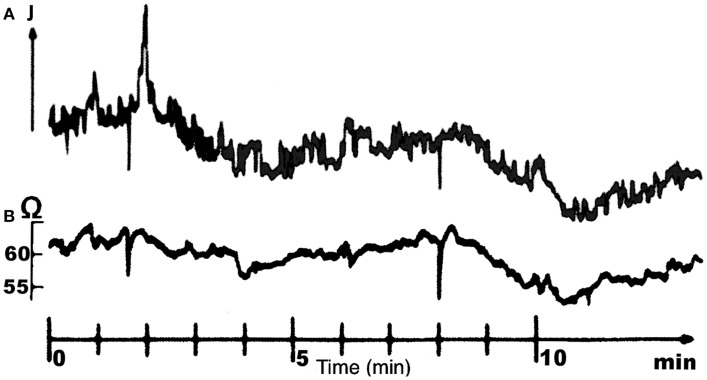
**Simultaneous recordings of the MOT spike frequency (A) and the light reflected by the anterior deep pseudopupil (DPP) (B) in the housefly**. It is clearly shown that the activity of MOT elicits a displacement of the DPP causing an angular shift of the photoreceptors’ optical axes. Adapted from (Hengstenberg, 1972).

Burtt and Patterson (1970) used antidromic light (Gemperlein and Järvilehto, 1969) to study the movement of the rhabdomeres. Other authors used a method consisted in examining the movements of the deep pseudopupil (DPP, Franceschini and Kirschfeld, 1971) elicited by angular shifts of the photoreceptors’ optical axes. Hengstenberg (1971, 1972) reported that the DPP movements were correlated with changes in the light intensity (see Figure 2), whereas Franceschini et al. (1991) recorded the activity of the MOT and the orbito-scapalis muscle (MOS) simultaneously in the walking fly and correlated these activities with the micrometric movements of the ipsilateral photoreceptors measured optically on the DPP (Franceschini et al., 1995; Franceschini and Chagneux, 1997). Franceschini et al. were the first to report the occurrence of large decreases (from 120 to 40 Hz) in the spike firing rates of the MOT and the MOS and a scanning amplitude in the order of 0.5–1Δφ, with Δφ the angle between two adjacent ommatidia (see review by Land, 1997). In addition, the spike firing rate decreases observed in the two muscles were not always synchronous, reflecting the complexity of the 2-D movements made by the photoreceptors. Similar periodic gaze shifts to the human micro-nystagmus (see review by Rolfs, 2009) have been observed in studies on the flying fly (Franceschini and Chagneux, 1997), where Franceschini and Chagneux reported that the frequency of the quasi-periodic scanning of the visual axes ranged approximately between 5 and 6 Hz. In a recent study on the fixed *Calliphora* blowfly, in which video analysis was combined with intracellular electrophysiological photoreceptor recordings, retinal movements of 0.35°(about 0.3Δφ) were found to be associated with periodic eye movements with a frequency of 5–7 Hz (Ciobanu et al., 2013).

### 2.2 Model for the fly’s eye muscle activity

Burtt and Patterson (1970) have reported that changes in the MOT firing rate can also be induced by moving a large striped pattern across the visual field. As discussed by Patterson (1973a), the level of illumination can affect the MOT activity and thus result, via a feedback mechanism, in the micro-scanning of the visual images by the photoreceptors. The following fundamental question was addressed by Qi and Northrop (1989) and Northrop (2001): how is eye muscle activity affected by visual stimulation? These authors investigated this question closely by placing a vertical stripe moving side to side in front of a fixed *Calliphora* fly while recording the MOT activity (action potentials). Based on the results obtained using several moving stimuli (sinusoidal and triangular displacement laws and even stepwise movements of the vertical stripe), Northrop and Qi concluded that the left and right MOT activities were apparently correlated with the speed of the moving stripe. This conclusion is not very surprising if one looks at the motion sensitive neurons of the fly (see the review by Taylor and Krapp, 2007). However, one might wonder what the point of a visual feedback loop may be for controlling the orientation of the visual axes (the gaze) in order to minimize the retinal slip speed. In addition, this closed-loop control system seems to depend on the fly’s ability to detect and measure angular target movements which are smaller than the interommatidial angle. In their study on the hoverfly’s flight behavior, Collett and Land concluded that binocular triangulation cannot be achieved by the male hoverfly because it would require a resolution equal to 1/40 of the interommatidial angle. This resolution was qualified by these authors as unrealistic. However, if one of the possible roles of these retinal micro-movements is to enhance the resolution, as found to be the case in many studies on artificial vibrating eyes (see Section “Bio-Inspired Visual Sensors Mimicking Animals’ Micro Eye Movements”), the occurrence of binocular triangulation would be perfectly feasible, and this might explain the vergence eye movements observed by Franceschini et al. (1991).

**Figure 3:**
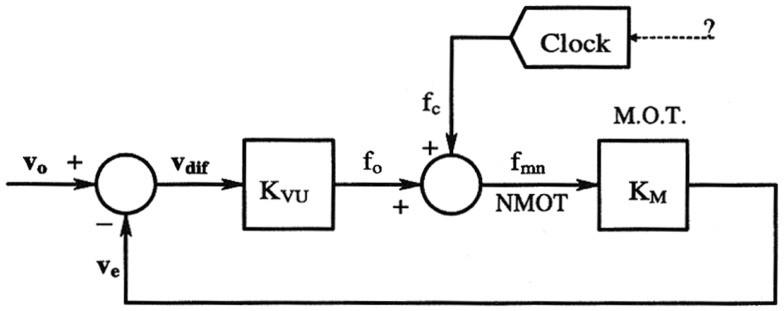
**Feedback-loop speed control system possibly involved in MOT activity**. The closed-loop would reduce the slip speed between the target motion (*v_o_*) and the angular speed of the visual axes (*v_e_*). In this model, the periodic response of the muscle is seen to be triggered by an external signal acting as an input disturbance on the visual feedback loop. *K_vu_* and *K_m_* are two pure gains, whereas the clock block can be regarded as a periodic signal generator. Reproduced with permission from Northrop (2001).

Although Zaagman et al. (1977) recorded responses of horizontally selective movement detectors to step displacements of a grating which were smaller than the interommatidial angle, no clear-cut evidence of motion hyperacuity (see Section “Some Findings on Visual Acuity”) has been obtained so far in the fly. However, many interesting questions arose as the result of Qi and Northrop’s experiments: these authors were the first to suggest that a closed-loop control system may be responsible for the eye muscle activity observed (see Figure 3). The exact role of this control system still remains to be determined, however.

## 3 Some Findings on Visual Acuity

As we will see in Section “Bio-Inspired Visual Sensors Mimicking Animals’ Micro Eye Movements”, artificial miniature eye movements and retinal micro-movements promote hyperacuity. At this stage, it is important to remember the fundamental difference existing between the following concepts:

- Hyperacuity: the ability to *locate* of a feature (such as contrasting bars or edges), regardless of its exact nature, with a greater accuracy than that corresponding to the resolution imposed by the photoreceptor’s limited pitch (Westheimer, 1981, 2009).
- Motion hyperacuity: the ability to *detect angular movements* smaller than the resolution imposed by the limitations of the optics (the interreceptor angle in the case of a camerular eye, or the interommatidial angle in that of a compound eye). Jumping spiders (Salticidae) are able, for example, to detect tiny displacements of a small target moving in the field of view of its anterior lateral eyes (Zurek and Nelson, 2012).
- Temporal hyperacuity: the ability to detect temporal disparities in the microsecond range, as occurs, for example, at the level of single neurons in electric fish (Kawasaki et al., 1988).
- Vernier acuity: the ability to distinguish between a closely spaced pair of lines. Hennig et al. have established mathematically that the human eye tremor can improve the spatial resolution and induce hyperacute responses to Vernier stimuli (Hennig and Wörgötter, 2004). In their fine model for human visual perception including the Gaussian receptive fields of the ganglion cells, Donner and Hemilä (2007) have established that micro-saccades can help to distinguish between closely spaced lines.

The various artificial vibrating eyes and bio-inspired aerial robots presented in Section “Bio-Inspired Visual Sensors Mimicking Animals’ Micro Eye Movements” are able to locate contrasting bars or edges with a greater accuracy than that imposed by the narrow interommatidial angle. These eyes can therefore be said to be genuine position sensing devices endowed with hyperacuity.

## 4 Bio-Inspired Visual Sensors Mimicking Animals’ Micro-Eye Movements

This active compound eye features two properties that are usually banned by optic sensor designers because they detract from the sharpness of the resulting images: optical blurring (see Stavenga, 2003 for review) and vibration (see Section “Retinal Micro-Movements in the Fly’s Compound Eye: A Review”). The active visual principle adopted in the various applications described in this section is based on a graded periodic back-and-forth eye rotation of a few degrees scanning the visual environment.

**Figure 4:**
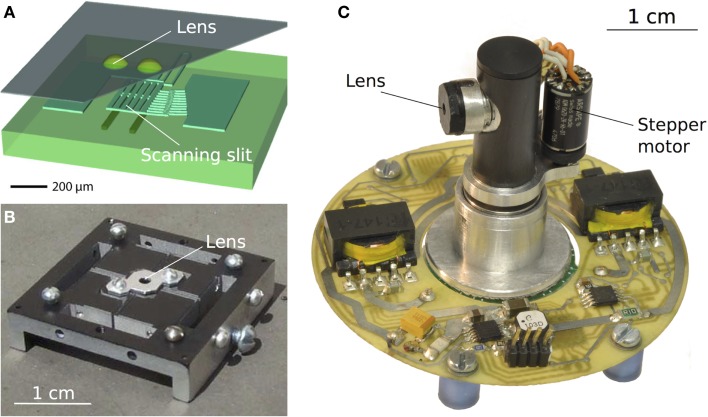
**(A)** A one-chip scanning sensor obtained using a photolithographic process. The back-and-forth movement of the visual axes was implemented here via an electrostatically driven scanning slit placed over the photodiodes [reproduced with permission from Hoshino et al. (2001)]. **(B)** A visual scanning sensor inspired by the spiders retinal movements. The passive scanning movements were powered here by environmental vibrations applied to the device (Landolt and Mitros, 2001). **(C)** Bio-inspired hyperacute vibrating eye composed of 6 pixels placed behind a fixed lens. The micro-scanning movement imposed to the whole eye was implemented by means of a tiny eccentric mechanism coupled to a small stepper motor (Juston and Viollet, 2012).

In 1996, Mura and Franceschini developed the first micro-scanning sensor based on the periodic retinal micro-movements observed in the fly (Mura and Franceschini, 1996). Thanks to its motion hyperacuity, this scanning eye was capable of detecting low levels of translational optic flow such as those encountered by a mobile robot around its heading direction (the focus of expansion). This was followed by the development of another micro-scanning visual sensor (Viollet and Franceschini, 1999a), which enabled a small aerial robot to locate a moving target, fixate it, and follow it smoothly (Viollet and Franceschini, 1999b, 2001). Several attempts (see Figures 4A–C) have been made to develop bio-inspired scanning sensors based on vibrating optic fibers (Mura and Shimoyama, 1998), a compound eye structure (Hoshino et al., 2000, 2001), passive structure (Landolt and Mitros, 2001), an actuated mirror (Landolt and Mitros, 2001), and even a tiny eccentric mechanism (Juston and Viollet, 2012). In these studies, an active visual process was mainly used to improve the detection of slowly moving targets. In the field of mechatronics, active movements applied to the optic fibers of a visual sensor have been used in several industrial applications to read bar codes (Yeatman et al., 2004).

**Figure 5:**
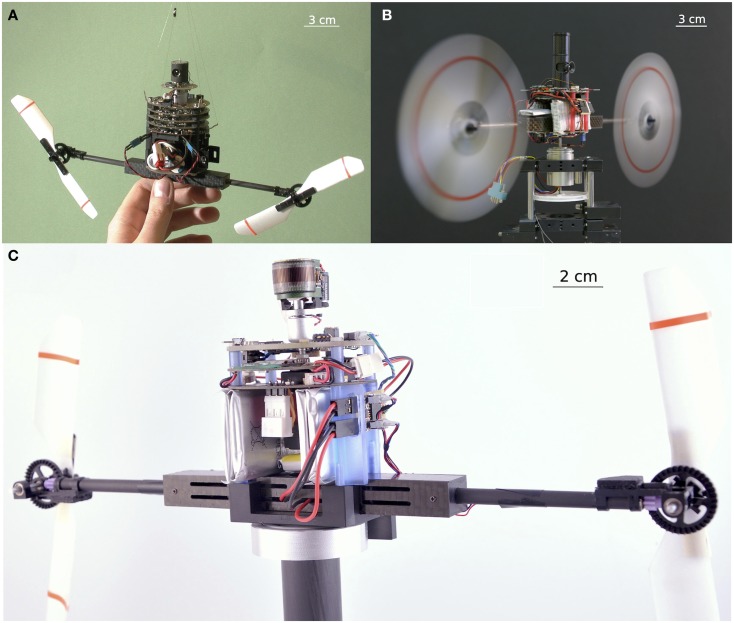
**Three generations of twin-rotor robots equipped with a vibrating eye inspired by the micro-movements of the fly’s retina (see Section “Retinal Micro-Movements in the Fly’s Compound Eye: A Review”)**. All these sighted robots are endowed with hyperacuity, i.e., they are able to locate and smoothly track a moving target with a much greater accuracy than that imposed by limitations of the pixel pitch of their eyes. **(A)** The 100-g OSCAR robot with an eye composed of only 2 pixels, scanning back and forth at a frequency of 10 Hz with an amplitude of 9° (Viollet and Franceschini, 1999a,b, 2001). **(B)** The VODKA robot equipped with its scanning eye, on which periodic micro-scanning movements were imposed by means of a piezo bender translating the two photodiodes placed behind a fixed lens (Kerhuel et al., 2007, 2010, 2012). The VODKA robot was able to locate a contrasting feature with a 900 times greater accuracy than its static optical resolution (without any micro-movements of the eye). **(C)** The HyperRob robot equipped with the active version of the artificial curved compound eye called CurvACE (Floreano et al., 2013).

Many visual sensors based on active retinal micro-movements have been used for various purposes, such as enhancing edge detection (Ando, 1988; Prokopowicz and Cooper, 1995; Hongler et al., 2003) and improving obstacle avoidance (Mura and Shimoyama, 1998). However, few studies have focused so far on the use of retinal vibrations to enhance visual acuity. Visual scanning at a variable angular speed was previously used to enhance the resolution by a factor of 40 in an edge-locating task (Viollet and Franceschini, 1999a,b), and more recently by a factor of 70 (Viollet and Franceschini, 2010). A pulsed-scanning mode was found to help a mobile robot detect the simple presence of edges in its visual field (Mura and Shimoyama, 1998). A circular micro-scanning mode was developed to improve the spatial resolution by transforming spatial information into temporal information (Landolt and Mitros, 2001). This same mode was also used to obtain line or edge operators by correlating a modulating signal with the output signals emitted by a 2-D imager (Ando, 1988). A recent study (Kerhuel et al., 2012) has focused on the processing of the amplitude of the photodector’s output signals. By applying sinusoidal micro-scanning movements to a retina composed of only 2 pixels, it was established that the ratio between the difference and the sum of the differentiated photodetector signals can lead to an outstanding degree of hyperacuity, which was 900 times higher than the interreceptor angle (2.87°). Figure 5 shows three generations of bio-inspired sighted aerial robotic platforms equipped with either an eye with a vibrating retina comprising only two pixels (Figures 5A,B) or an artificial compound eye (Floreano et al., 2013) subjected to a periodic micro-scanning movement. All these robots are endowed with hyperacuity, and they can lock their gaze onto a moving contrasting target (bars or edges) and track it smoothly by automatically controlling the orientation of their eye (in the case of the robots shown in Figures 5B,C) and thus, the orientation of their body (their heading). Table 1 summarizes the features of the different scanning sensors inspired by the fly’s retinal micro-movements and the benefits of the visual scanning in terms of optical resolution enhancement.

**Table 1:** **Summary of bio-inspired visual scanning sensors**.

Scanning technique	Reference	Scanning amplitude (°)	Scanning frequency (Hz)	Optical resolution without/**with** scanning (°)	Benefit of the scanning
Galvanometer	Mura and Franceschini (1996)	6	50	3	Detection low optic flow
Magnetic coil	Mura and Shimoyama (1998)	6	7	3	Motion hyperacuity
Servomotor	Viollet and Franceschini (1999a)	8	10	5/**0.05**	Hyperacuity
Piezo actuator	Hoshino et al. (2001)	7	5	3.2	Motion hyperacuity
Scanning slit (electrostatic)	Hoshino et al. (2001)	3	5–20	5/**0.5**	Hyperacuity
Piezo actuator	Viollet and Franceschini (2010)	5.6	10	2.8/**0.04**	Hyperacuity
Piezo actuator	Kerhuel et al. (2012)	0.2	40	2.87/**0.003**	Hyperacuity
Piezo actuator	Juston et al. (2014)	8.4	50	4.2/**0.025**	Hyperacuity

*Whenever a visual sensor was endowed with hyperacuity, its dynamic resolution (with scanning) is given in bold*.

Finally, the authors of another recent study (Juston et al., 2011) have shown that micro-movements of this kind can enable a visual scanning sensor to locate the horizontal roof of a distant building (56 m) with a resolution (0.025°), which is at least 160-fold greater than the sensors static resolution (4°). In addition, phase analysis of the modulated visual signals led to the development of a novel edge-bar detector (Juston et al., 2014): it turned out that if the contrasting feature is a bar, the phase difference between the two modulated signals will be 180°, whereas in the case of an edge, it will be null (see Juston et al., 2014 for further details).

## 5 Suggestions for Future Research

**Figure 6:**
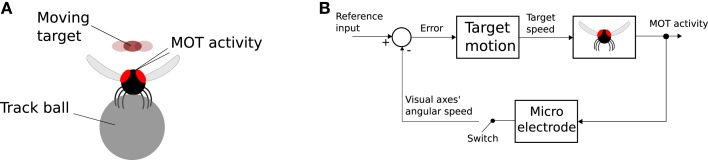
**(A)** Simplified diagram of a fixed fly walking on a track ball while its MOT response to the laterally to-and-fro moving target placed in front of it is recorded. **(B)** Closed-loop control of the target’s speed depending on the MOT activity. If the MOT control system is correlated with the speed of the target, then the latter will remain stationary regardless of the speed reference input signal. In the closed-loop scheme presented here, a second feedback-loop has been added to that proposed by Northrop and Qi (see Figure 3): in this case, the input signal is the target speed and the output signal is the angular speed of the visual axes. The switch makes it possible to open or close the feedback loop, depending on the experimental procedure used.

It would be interesting to check the MOT responses of a fixed walking fly placed in front of a moving target, the linear position of which is controlled in a closed-loop mode (see Figure 6). As described by Northrop and Qi (Northrop, 2001), a target moving laterally to-and-fro in front of a walking fly triggers MOT activity, which is correlated with the speed of the target. Under the closed-loop conditions presented in Figure 6B, the target’s motion will be controlled by the error between a reference input signal (a sinusoidal signal, for example) and the angular speed of the visual axes estimated by recording the MOT activity. Therefore, if the rotation of the visual axes faithfully follows the motion imposed on the target, the feedback loop controlling the speed described in Figure 6B will completely immobilize the target. It is assumed here that the MOT activity is scaled to match the angular speed of the visual axes. This scaling can be applied via the DPP. It is also assumed that the time required to make the target move in response to any change in the MOT activity is very short.

In the fixed walking fly, it has been established that the MOT activity is quasi periodic and that it has a much larger amplitude (see Franceschini and Chagneux, 1997) than that recorded in a stationary fixed fly. In addition, it might be worth using the setup described in Figure 6A to test the responses of motion sensitive neurons (such as the H1-cells) to step displacements smaller than the interommatidial angle, while concomitantly recording the MOT activity (as suggested by Zaagman in *Calliphora*, see Zaagman et al., 1977).

## 6 Summary

The present review deals with studies dating back to the 70s on the fly’s retinal micro-movements and the development of bio-inspired visual sensors involving similar miniature eye movements. Although no direct connection between retinal eye movements and visual hyperacuity has yet been clearly established, several experiments on humans and animals tend to prove that this active visual process may improve the resolution well beyond the static resolution imposed by the limitations of the optics. Studies on retinal movements and miniature robotic eye movements (micro-saccades) have not yet brought to light all the possible means of improving the detection and localization of contrasting features. A great deal of research is still required before it will be possible to specify the role of micro-eye movements, which cannot simply be reduced to a means of preventing vision from fading away. Some extremely interesting paths of investigation have already been opened, however, in the fields of biology and robotics, where it has been established, for example, that the accuracy of localization performances can be improved 900-fold in comparison with what can be achieved using the low resolution imposed by the optics (see Section “Bio-Inspired Visual Sensors Mimicking Animals’ Micro Eye Movements”). There is still room for new approaches which may lead to the development of innovative sensing devices such as visual odometers and visual sensors dedicated to tracking moving 2-D targets in a natural environment. As a fair return, there is also room for fundamental research projects in which the validity of biological hypotheses can be tested on man-made machines and sensors.

## Conflict of Interest Statement

The author declares that the research was conducted in the absence of any commercial or financial relationships that could be construed as a potential conflict of interest.

## References

[B1] Ahissar E. Arieli A. (2012). Seeing via miniature eye movements: a dynamic hypothesis for vision. Front. Comput. Neurosci. 6:89 10.3389/fncom.2012.00089 23162458 PMC3492788

[B2] Ando S. (1988). “Texton finders based on Gaussian curvature of correlation with an application to rapid texture classification,” in Proc. IEEE International Conference on Systems, Man, and Cybernetics, Vol. 1 (Beijing and Shenyang: IEEE), 25–28

[B3] Burrows M. Horridge G. A. (1968). The action of the eyecup muscles of the crab, carcinus, during optokinetic movements. J. Exp. Biol. 49, 223–250

[B4] Burtt E. T. Patterson J. A. (1970). Internal muscle in the eye of an insect. Nature 228, 183–184 10.1038/228183a0 5460025

[B5] Carpenter R. H. S. (1988). Movements of the Eyes: Miniature Movements, 2nd Edn, Chap. 6. London: PION

[B6] Ciobanu L. G. Wiederman S. D. O’Carroll D. C. (2013). Retinal movements in the blowfly *Calliphora stygia*. Front. Physiol. 10.3389/conf.fphys.2013.25.00120

[B7] Collett T. S. Land M. F. (1975). Visual control of flight behaviour in the Hoverfly *Syritta pipiens L*. J. Comp. Physiol. A Neuroethol. Sens. Neural. Behav. Physiol. 99, 1–66

[B8] De Rossi D. E. Pieroni M. (2013). Grand challenges in bionics. Front. Bioeng. Biotechnol. 1 10.3389/fbioe.2013.00003 PMC4090898 25023011

[B9] Donner K. Hemilä S. (2007). Modelling the effect of microsaccades on retinal responses to stationary contrast patterns. Vision Res. 47, 1166–1177 10.1016/j.visres.2006.11.024 17368501

[B10] Floreano D. Pericet-Camara R. Viollet S. Ruffier F. Brückner A. Leitel R. (2013). Miniature curved artificial compound eyes. Proc. Natl. Acad. Sci. U.S.A. 110, 9267–9272 10.1073/pnas.1219068110 23690574 PMC3677439

[B11] Franceschini N. Chagneux R. (1997). “Repetitive scanning in the fly compound eye,” in Göttingen Neurobiology Report, Vol. 2 eds Wässle H. Elsner N. (Göttingen: Thieme), 279

[B12] Franceschini N. Chagneux R. Kirschfeld K. (1995). “Gaze control in flies by coordinated action of eye muscle,” in Göttingen Neurobiology, Vol. 2, 402

[B13] Franceschini N. Chagneux R. Kirschfeldand K. Mücke A. (1991). “Vergence eye movements in flies,” in Göttingen Neurobiology Report, Vol. 1, (Göttingen: Thieme), 275

[B14] Franceschini N. Kirschfeld K. (1971). Etude optique in vivo des éléments photorécepteurs dans l’oeil composé de *Drosophila*. Biol. Cybern. 8, 1–13 10.1007/BF00270828 5558428

[B15] Gemperlein R. Järvilehto M. (1969). Direkte beobachtung der rhabdomere bei *Calliphora erythrocephala* (Meig.). Z. Vergl. Physiologie 65, 445–454 10.1007/BF00299053

[B16] Hengstenberg R. (1971). Das augenmuskelsystem der stubenfliege musca domestica. Kybernetik. 9, 56–77 10.1007/BF00270852 5566799

[B17] Hengstenberg R. (1972). “Eye movements in the housefly musca domestica,” in Information Processing in the Visual Systems of Anthropods, ed. Wehner R. (Berlin: Springer), 93–96

[B18] Hennig M. H. Wörgötter F. (2004). “Eye micro-movements improve stimulus detection beyond the nyquist limit in the peripheral retina,” in NIPS, (Vancouver: MIT press), 16

[B19] Hongler M. O. de Meneses Y. L. Beyeler A. Jacot J. (2003). The resonant retina: exploiting vibration noise to optimally detect edges in an image. IEEE Trans. Pattern Anal. Mach. Intell. 25, 1051–1062 10.1109/TPAMI.2003.1227982

[B20] Hoshino K. Mura F. Shimoyama I. (2000). Design and performance of a micro-sized biomorphic compound eye with a scanning retina. J. Microelectromech. Syst. 9, 32–37 10.1109/84.825774

[B21] Hoshino K. Mura F. Shimoyama I. (2001). A one-chip scanning retina with an integrated micromechanical scanning actuator. J. Microelectromech. Syst. 10, 492–497 10.1109/84.967370

[B22] Juston R. Kerhuel L. Franceschini N. Viollet S. (2014). Hyperacute edge and bar detection in a bioinspired optical position sensing device. IEEE ASME Trans. Mechatron. 9:1025–34 10.1109/TMECH.2013.2265983

[B23] Juston R. Viollet S. (2012). “A miniature bio-inspired position sensing device for the control of micro-aerial robots,” in 2012 IEEE/RSJ International Conference on Intelligent Robots and Systems (IROS) (Villamora: IEEE), 1118–1124

[B24] Juston R. Viollet S. Kerhuel L. Franceschini N. (2011). High performance optical angular position sensing at low-cost: a bio-inspired approach. Sensors, 2011 IEEE, 378–381 10.1109/ICSENS.2011.6127151

[B25] Kaps F. Schmid A. (1996). Mechanism and possible behavioural relevance of retinal movements in the ctenid spider *Cupiennius salei*. J. Exp. Biol. 199(Pt 11), 2451–2458 9320378 10.1242/jeb.199.11.2451

[B26] Kawasaki M. Rose G. Heiligenberg W. (1988). Temporal hyperacuity in single neurons of electric fish. Nature 336, 173–176 10.1038/336173a0 2847057

[B27] Kerhuel L. Viollet S. Franceschini N. (2007). “A sighted aerial robot with fast gaze and heading stabilization,” in 2007 IEEE/RSJ International Conference on Intelligent Robots and Systems (IROS) (San Diego: IEEE), 2634–2641

[B28] Kerhuel L. Viollet S. Franceschini N. (2010). Steering by gazing: an efficient biomimetic control strategy for visually guided micro aerial vehicles. IEEE Trans. Robot. 26, 307–319 10.1109/TRO.2010.2042537

[B29] Kerhuel L. Viollet S. Franceschini N. (2012). The vodka sensor: a bio-inspired hyperacute optical position sensing device. IEEE Sens. J. 12, 315–324 10.1109/JSEN.2011.2129505

[B30] Ko H.-K. Poletti M. Rucci M. (2010). Microsaccades precisely relocate gaze in a high visual acuity task. Nat. Neurosci. 13, 1549–1553 10.1038/nn.2663 21037583 PMC3058801

[B31] Kuang X. Poletti M. Victor J. D. Rucci M. (2012). Temporal encoding of spatial information during active visual fixation. Curr. Biol. 22, 510–514 10.1016/j.cub.2012.01.050 22342751 PMC3332095

[B32] Kuiper J. W. Leutscher-Hazelhoff J. T. (1965). High-precision repetitive firing in the insect optic lobe and a hypothesis for its function in object location. Nature 206, 1158–1160 10.1038/2061158b0 5866159

[B33] Land M. (1982). Scanning eye movements in a heteropod mollusc. J. Exp. Biol. 96, 427–430

[B34] Land M. F. (1969). Movements of the retinae of jumping spiders (Salticidae: Dendryphantinae) in response to visual stimuli. J. Exp. Biol. 51, 471–493 5351426 10.1242/jeb.51.2.471

[B35] Land M. F. (1997). Visual acuity in insects. Annu. Rev. Entomol. 42, 147–177 10.1146/annurev.ento.42.1.147 15012311

[B36] Landolt O. Mitros A. (2001). Visual sensor with resolution enhancement by mechanical vibrations. Auton. Robots 11, 233–239 10.1023/A:1012482822516

[B37] Martinez-Conde S. Otero-Millan J. Macknik S. L. (2013). The impact of microsaccades on vision: towards a unified theory of saccadic function. Nat. Rev. Neurosci. 14, 83–96 10.1038/nrn3405 23329159

[B38] Mura F. Franceschini N. (1996). “Obstacle avoidance in a terrestrial mobile robot provided with a scanning retina,” in Proc. IEEE Intelligent Vehicles Symposium (Tokyo: IEEE), 47–52

[B39] Mura F. Shimoyama I. (1998). “Visual guidance of a small mobile robot using active, biologically-inspired, eye movements,” in Proc. IEEE International Conference on Robotics and Automation, Vol. 3 (Leuven: IEEE), 1859–1864

[B40] Northrop R. B. (2001). “Large arrays of interacting receptors: the compound eye,” in Introduction to Dynamic Modeling of Neuro-Sensory Systems, ed. Neuman (Boca Raton: CRC Press), 298–305

[B41] Patterson J. A. (1973a). The eye muscle of *Calliphora vomitoria* L: II. Transient responses to changes in the intensity of illumination. J. Exp. Biol. 58, 585–598

[B42] Patterson J. A. (1973b). The eye muscle of *Calliphora vomitoria* L: I. Spontaneous activity and the effects of light and dark adaptation. J. Exp. Biol. 58, 565–583

[B43] Prokopowicz P. N. Cooper P. R. (1995). The dynamic retina: contrast and motion detection for active vision. Int. J. Comput. Vis. 16, 191–204 10.1007/BF01539626

[B44] Qi X. Northrop R. (1989). “Dynamic properties of the clock-spike system of the fly,” in Images of the Twenty-First Century, Proceedings of the Annual International Conference of the IEEE Engineering in Medicine and Biology Society, Vol. 5 (Seatle: IEEE), 1678–1679

[B45] Rolfs M. (2009). Microsaccades: small steps on a long way. Vision Res. 49, 2415–2441 10.1016/j.visres.2009.08.010 19683016

[B46] Sandeman D. C. (1978). Eye-scanning during walking in the crab *Leptograpsus variegatus*. J. Comp. Physiol. A Neuroethol. Sens. Neural. Behav. Physiol. 124, 249–257 10.1007/BF00657056

[B47] Stavenga D. G. (2003). Angular and spectral sensitivity of fly photoreceptors. I. Integrated facet lens and rhabdomere optics. J. Comp. Physiol. A Neuroethol. Sens. Neural. Behav. Physiol. 189, 1–17 12548425 10.1007/s00359-002-0370-2

[B48] Taylor G. K. Krapp H. G. (2007). “Sensory systems and flight stability: What do insects measure and why?,” in Insect Mechanics and Control, volume 34 of Advances in Insect Physiology, eds Casas J. Simpson S. (Academic Press), 231–316

[B49] Viollet S. Franceschini N. (1999a). “Biologically-inspired visual scanning sensor for stabilization and tracking,” in 1999 IEEE/RSJ International Conference on Intelligent Robots and Systems IROS ’99. Proceedings, Vol. 1 (Kyongju: IEEE), 204–209

[B50] Viollet S. Franceschini N. (1999b). “Visual servo system based on a biologically inspired scanning sensor,” in Sensor Fusion and Decentralized control in Robotics II, Vol. 3839 eds McKee G. T. Schenker P. S. (Boston: SPIE), 144–155

[B51] Viollet S. Franceschini N. (2001). “Super-accurate visual control of an aerial minirobot,” in Autonomous Minirobots for Research and Edutainment, AMIRE, ed. Witkowski M. (Paderborn: Heinz Nixdorf Institute), 215–224

[B52] Viollet S. Franceschini N. (2010). A hyperacute optical position sensor based on biomimetic retinal micro-scanning. Sens. Actuators A Phys. 160, 60–68 10.1016/j.sna.2010.03.036

[B53] Webb B. (2000). What does robotics offer animal behaviour? Anim. Behav. 60, 545–558 10.1006/anbe.2000.1514 11082225

[B54] Westheimer G. (1981). Visual Hyperacuity. Sensory Physiology 1. Berlin: Springer-Verlag

[B55] Westheimer G. (2009). Hyperacuity. Encyclopedia Neurosci. 5, 45–50 10.1016/B978-008045046-9.00218-7

[B56] Yeatman E. M. Kushner P. J. Roberts D. A. (2004). Use of scanned detection in optical position encoders. IEEE Trans. Instrum. Meas. 53, 37–44 10.1109/TIM.2003.821502

[B57] Zaagman W. Mastebroek H. Buyse T. Kuiper J. (1977). Receptive field characteristics of a directionally selective movement detector in the visual system of the blowfly. J. Comp. Physiol. 116, 39–50 10.1007/BF00605515

[B58] Zurek D. B. Nelson X. J. (2012). Hyperacute motion detection by the lateral eyes of jumping spiders. Vision Res. 66, 26–30 10.1016/j.visres.2012.06.011 22750020

